# Enabling Transdisciplinary Collaboration: Stakeholder Views on Working With “Children With Mentally Ill Parents” Research Groups

**DOI:** 10.3389/fpsyt.2021.760716

**Published:** 2021-11-23

**Authors:** Raphaela E. Kaisler, Christiane Grill

**Affiliations:** LBG Open Innovation in Science Center, Ludwig Boltzmann Gesellschaft (LBG), Vienna, Austria

**Keywords:** transdisciplinary collaboration, patient and public involvement, stakeholder engagement, open innovation, children of parents with a mental illness (COPMI), mixed-methods design, transdisciplinary research teams

## Abstract

Working collaboratively and openly together with stakeholders has become a common phenomenon in research. While previous studies have gathered a clear picture on researchers' attitudes, motivations, and barriers for actively involving stakeholders in transdisciplinary research, the stakeholder perspective is yet unknown. Therefore, this paper sets out to identify how stakeholders perceive transdisciplinary collaborations with researchers. This paper in particular reveals the enablers and barriers for such collaborations from the viewpoint of stakeholders. To do so, we look at how stakeholders, who were actively involved in the governance structure of two “children with mentally ill parents” research groups in Austria, perceived their collaboration with researchers. We used a mixed-method, quantitative-qualitative design. We conducted an online survey and interviews with the members of the advisory board and competence group. These stakeholders reported great satisfaction with the transdisciplinary collaboration and emphasized the value of different expertise. As the most important enablers for successful, transdisciplinary collaboration stakeholders emphasized researchers' open-mindedness toward new perspectives and approaches, flexibility to adapt to the research process along the way, and creativity dealing with diverse backgrounds and skills. Stakeholders further underlined the importance of a person facilitating the collaboration process between researchers and stakeholders to resolve any tensions and insecurities. Concluding, researchers' attitudes, and in particular their understanding of the value of stakeholder involvement in research are key enablers for successful transdisciplinary research collaborations.

## Introduction

The prevalence of children who live with a parent having a mental illness (COPMI) is about 25% worldwide (1–4). These children are at increased risk of long-term difficulties due to genetic, individual, family, and environmental risk factors (1, 5–8). Specific interventions related to family, social support and community have been shown to make a difference to the selected target groups (children, parents) and settings [psychiatry, community; (9–13)]. Research identified several external factors, governing service practices and the context itself as enablers for a successful implementation of COPMI interventions and services (14–17). Specifically regarding the context, professional influences (i.e., formal and informal norms, rules, policies, standards guiding the professionalization of individuals involved in the implementation) and the social climate (i.e., beliefs, values, customs and practices of the larger community, the system within which the intervention is embedded) are essential. For example, Zeichmeister-Koss et al. (18) recently analyzed the situational context and services of COPMI in the pilot region Tyrol in Austria. The authors found a lack of support processes and standards to meet children's needs and highlighted the gap between research and practice in the Austrian COPMI field.

This gap is not specific to the COPMI field. It generally points to a problem of inter- and transdisciplinary collaborations between researchers, stakeholders, and service user groups [e.g., patients, children and their families; (19)]. Interdisciplinary and transdisciplinary research performance and evaluation are both generative processes of harvesting, capitalizing, and leveraging multiple expertise (20). Here, we distinguish between interdisciplinary research as collaboration between different research disciplines, and transdisciplinary research as work between different research disciplines and stakeholders, such as practitioners, clinicians, patients, people with lived experience in mental illness and health, and family members.

In this article, we now describe and reflect how stakeholders perceive transdisciplinary collaborations with researchers. We analyze the enablers and barriers for such collaborations from the viewpoint of stakeholders. To do so, we look at how stakeholders who were actively involved in the governance structure of two COPMI research groups in Austria perceived their collaboration with researchers.

### Perceptions of Transdisciplinary Collaboration

Working collaboratively and openly together with stakeholders across transdisciplinary boundaries has become a common phenomenon in research (21). In the last few years, the importance of involving patients and other stakeholders in health-related research has steadily been growing in the UK (22) as well as worldwide (23–25). Patient and public involvement (PPI) refers to meaningful and active involvement of patients and members of the public in research activities and processes. Consequently, research is carried out “with” or “by” members of the public rather than “to”, “about”, or “for” them (22, 26). “By involving patients in their research, researchers learn from other people's experience, which then changes their own thinking, values, choices, and actions. This leads to the commonly reported outcomes of involvement—improved research design, delivery, and dissemination—and over time, the wider impacts of a changed research culture and agenda (27).” Public involvement in health-related research has shown that patients and members of the public are indeed able to successfully contribute to specific research problems as well as able to find innovative solutions, for example, via setting research priorities (28), co-producing knowledge (29, 30) or via shaping health care services (31). In line with this, several systematic reviews (32–36) have reported that stakeholder involvement makes a difference to the people affected. However, this type of involvement is also criticized of being weak and anecdotal. Criticism has particularly focused on the lack of empirical data to evaluate impact, the insufficient attention that is paid to the context in which involvement takes place, and the way involvement is actually lived (37). To counteract this criticism and ultimately to avoid tokenistic involvement of stakeholders in research, it is therefore crucial to determine “why” and “who” should be involved at all in research and to acknowledge the experiential knowledge that stakeholders bring to the table. In doing so, active involvement of stakeholders in research may ultimately maximize the opportunities of learning, increase the likelihood of impact, and help to achieve the goal of improved services to the affected community (38).

How researchers perceive transdisciplinary research by involving patients and the public has already been well-studied. Several studies have analyzed researchers' attitudes and motivations for working transdisciplinary with stakeholders. While researchers highlighted the potential benefits of involving the public, they yet expressed strong ambivalence regarding the exact purpose and value of patient and public involvement (19, 39, 40). Furthermore, a few studies have also assessed researchers' viewpoints regarding the barriers that hinder transdisciplinary stakeholder involvement. These studies identified a mix of barriers; particularly, lack of funding, time, and skills, finding the “right” people, organizational and policy barriers, research fatigue, group dynamics (41, 42), researchers' negative attitudes toward PPI (43) and personality characteristics (44). In a recent systematic review of reviews, Ocloo et al. (45) summarized various enablers and barriers of PPI in health and social research from the viewpoint of researchers. These were personal/individual factors, patient/relative involvement and attitudes, health professional relationships with patients, clarity of roles and expectations, knowledge, information and communication, financial compensation and resources, training, general support, power dynamics and organizational constraints, recruitment, and community approach.

How patients and the public perceive transdisciplinary collaboration with researchers is, however, unclear. Thus far, there exists no study analyzing how stakeholders involved in health research view their involvement in research. Therefore, this paper sets out to identify how stakeholders perceive transdisciplinary collaborations with researchers. We are hereby in particular interested in exploring the enablers and barriers of transdisciplinary research collaboration from a stakeholder perspective.

### Our Transdisciplinary Collaboration Approach in the Copmi Field

In a first step, the Ludwig Boltzmann Gesellschaft (LBG) launched the crowdsourcing project “Tell Us! What Questions about Mental Illness Should Science Take Up?” (46). The entire health care community in Austria (i.e., patients, family members, and health care professionals) was invited to submit research questions for the field of mental health. After analyzing and thematically collating 400 high-quality submissions, 17 topics were distilled. Out of these 17 topics, a focus on “children of mentally ill parents” (COPMI) emerged as the top research priority. Based on this outcome, 136 PhD students and post-doctoral researchers were invited to an “Ideas Lab”: 29 researchers participated in an “Ideas Lab on COPMI” (47). Two people with lived experience were invited to the Ideas Lab to share their experience as children of mentally ill parents, and to ultimately inspire researchers for future research. As an outcome of the Ideas Lab, two research groups “DOT—The Open Door” (48) and “Village—How to Raise a Village to Raise a Child” (49) were established. “DOT” focuses on early adolescents making the difficult leap from primary to secondary school and how supportive relationships between peers help children stay mentally and physically healthy. “Village” aims to strengthen formal and informal support structures around the child through enhancing their village of collaborative care. A relationship manager supported the research groups to establish community and stakeholder interactions, foster patient and public involvement activities, and to accompany them over the 4-year funding period (in total six million Euro).

To ensure transdisciplinary collaboration with stakeholders, the LBG introduced a novel governance structure for the two research groups. Two advisory groups and a competence group consisting of COPMI stakeholders were established for the two research groups. The advisory board each consisted of three scientific experts from different fields (e.g., psychiatry, psychology, implementation science, linguistics, gamification), two adults who lived with a parent with mental illness in their childhood, and an open innovation expert. The advisory boards discussed the research groups' achievements as well as their outlook for the future. In total, the advisory board met six times over the period of 4 years. Due to the COVID-19 pandemic, half of the meetings were held online in 2020 and 2021 via the Zoom video conferencing platform. The competence group consisted of five people (20–30 years old) who lived with a parent with mental illness in their childhood and had various professional backgrounds (e.g., in social work, art, public health, education). The competence group received an honorarium for their contributions and met on average 10 times a year to advice on the research groups' project design, methods, results, and dissemination strategies. The meetings were shifted online in 2020 and 2021 due to the COVID-19 pandemic.

### Aim of This Study

In this study, we analyze how stakeholders who were involved as advisory board or competence groups members in the two research groups “DOT—The Open Door” and “Village—How to Raise the Village to Raise the Child” perceive transdisciplinary collaborations with researchers. We are interested in stakeholder views since their perspective on transdisciplinary collaboration has been neglected in health-related research thus far. Furthermore, we identify enablers and barriers for transdisciplinary collaborations between researchers and stakeholders. Therefore, this study sets out to answer the following two research questions: How do stakeholders perceive transdisciplinary collaboration with researchers? What are the enablers and barriers for successful, transdisciplinary research collaborations?

## Methods

To answer the two research questions, we used a mixed-methods, quantitative-qualitative design. First, all advisory board and competence group members were asked to fill in a questionnaire. This first step aimed to reveal the stakeholders' general perceptions of transdisciplinary collaboration. In a subsequent step, we wanted to gain more in-depth insights, thoughts, and reasons of the stakeholders involved in research. Therefore, we conducted semi-structured interviews with purposefully selected advisory board and competence group members.

### Survey

#### Participants

All 13 advisory board members (thereof three males) and all six competence group members (all females) of the two research groups “DOT—The Open Door” and “Village—How to Raise the Village to Raise the Child” were invited to fill in an online survey.

#### Procedure

The questionnaire was designed with the online survey tool Unipark^®^ (Tivian). An anonymously link to the survey was sent to the members via a personalized email explaining the objective and rationale of the study and asking them to complete a 7-min-long survey. The survey link was open for 6 weeks from April 8 to May 18, 2021. Various reminders were sent via email throughout the 6 weeks. Responses to the survey were then quantitatively analyzed.

#### Measures

After agreeing to the informed consent, respondents were asked a range of closed-ended questions and one open-ended question. Questions addressed the following themes: the general setup of the meetings (e.g., frequency, preparation material), the structure of the advisory and competence groups (e.g., different expertise), the quality of involvement (e.g., atmosphere, contributions), and the collaboration with researchers. To measure each theme thoroughly, two to six statements were formulated for each theme and respondents were asked to indicate their agreement with each statement along a 5-point Likert scale (1 = do not agree at all −5 = fully agree). Respondents were also asked about their overall satisfaction with the structure of the advisory boards and competence groups and the development of the research group (5-point Likert scale: 1 = not at all satisfied −5 = fully satisfied). Respondents were also asked in how far they would recommend others to participate in such advisory boards and competence groups (5-point Likert scale: 1 = not at all recommended −5 = very much recommended). Lastly, in an open-ended question, respondents were asked about their overall impression of their work.

### Interviews

#### Participants

Four advisory board members (one adult who lived with a parent with mental illness in their childhood, one expert in open innovation in science, two experts from the field of psychology) and two competence group members (two adults who lived with a parent with mental illness in their childhood) were asked to be interviewed. Interviewees were selected based on their role in the advisory board and competence group. Among the invited interviewees were two men and four women.

#### Procedure

Questions for a 1-h long, semi-structured interview were designed and personalized invitation emails explaining the objective and rationale of the interview were sent out. Prior to the interview, the interviewer explained the procedure to the interviewees and obtained written, informed consent in accordance with the ethical guidelines in Austria and the Declaration of Helsinki. All interviewees gave informed consent to be recorded and to publish the data. All interviews were then held online via Zoom and transcribed. The transcripts were then anonymized: all identifying information was removed from the transcripts. The data was then analyzed using thematic analysis.

#### Interview Guide

The semi-structured interviews covered a range of different topics. These were the interviewee's role in the advisory board or competence group, the collaboration with the researchers (particularly, the joint development of approaches, the integration of different perspective, the challenges for researchers, and differences to rather traditional approaches), and the enablers and barriers for successful, transdisciplinary collaboration.

## Results

### Survey

Nine advisory board members (response rate: 69%) and three competence group members (response rate: 50%) completed the online survey. Due to the small sample size, we conducted a descriptive, univariate analysis. Here, we report the means (M) and standard deviations (SD) for each survey item (Supplementary Table 1).

**Figure 1:**
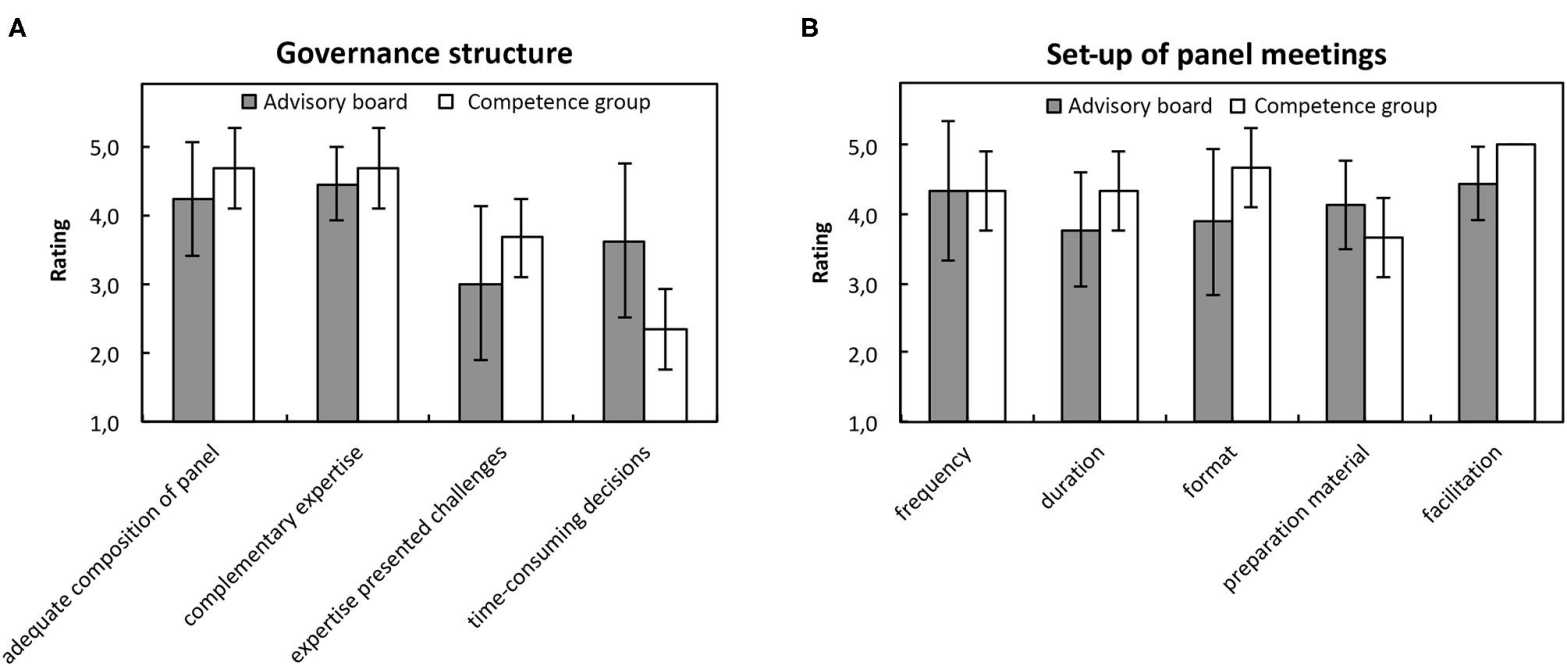
Structure of the panel meetings. **(A)** Shows average ratings of the governance structure and **(B)** the set-up of panel meetings in the online survey. Gray boxes indicate ratings of the advisory board members and white boxes indicate ratings of the competence group members.

As to the general setup of the meetings, both, members of the advisory board and members of the competence group, assessed the frequency (M = 4.3, SD = 0.9), duration (M = 3.9, SD = 0.8), format (M = 4.1, SD = 1.0), preparation material (M = 4.0, SD = 0.6), and particularly the facilitation (M = 4.6, SD = 0.05) of the meetings very positively (Figure 1A).

Overall, respondents rated the structure of the advisory board and competence group as adequate (Figure 1B). Specifically, the composition of these two panels was very adequate for the research groups (M = 4.3, SD = 1.0), and the different expertise on the panels very well-complemented each other (M = 4.5, SD = 0.5). Simultaneously, however, the different expertise among the members of the panels presented challenges (M = 3.2, SD = 1.0) and resulted in more time-consuming decisions (M = 3.3, SD = 1.0). Particularly, the members of the competence group assessed the challenges (M = 3.7, SD = 0.6) and time consumption (M = 3.6, SD = 0.6) of their work due to different expertise slightly more critically than the members of the advisory board (challenges: M = 3.0, SD = 1.1, time: M = 2.3, SD = 0.6).

**Figure 2:**
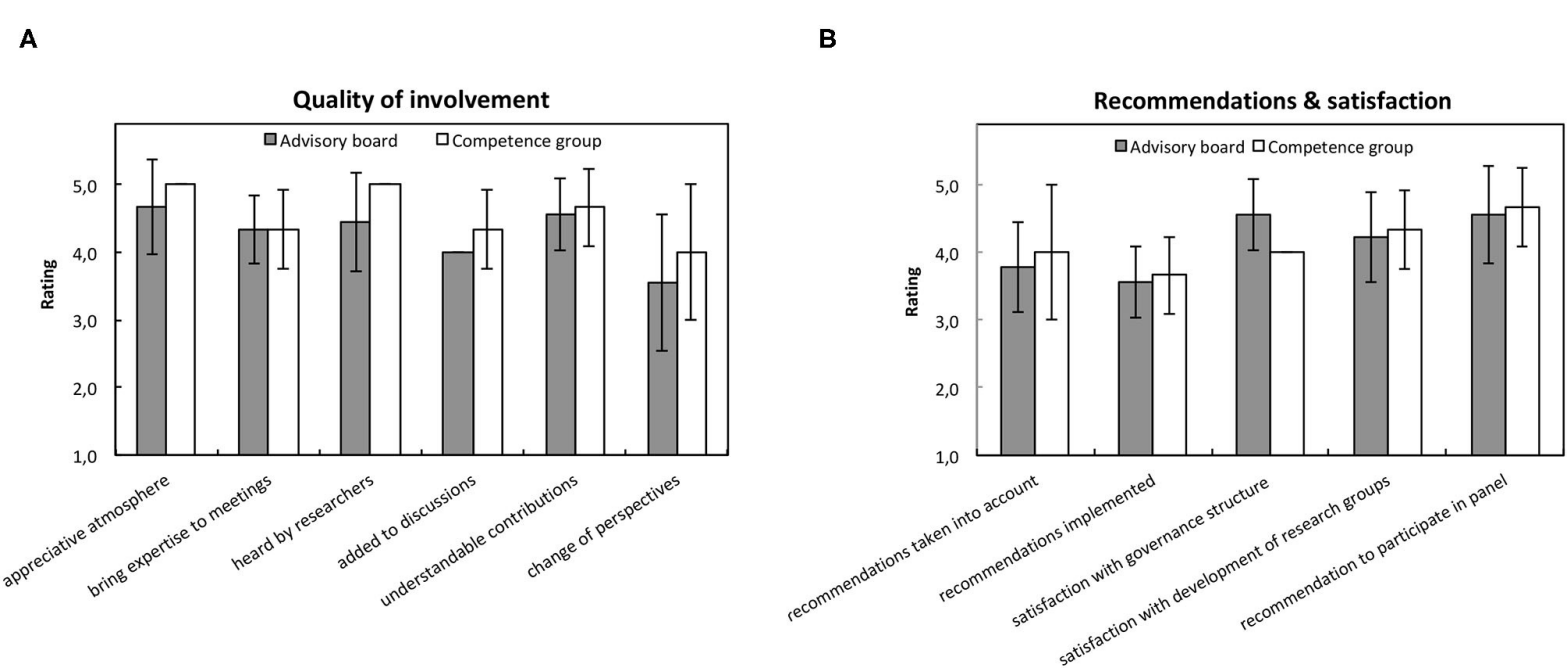
Quality of involvement in the panel meetings. **(A)** Shows average ratings of the quality of involvement and **(B)** recommendations and satisfaction with the panel meetings in the online survey. Gray boxes indicate ratings of the advisory board members and white boxes indicate ratings of the competence group members.

Moreover, the quality of involvement was rated positively (Figure 2A). The atmosphere in the panels was appreciative (M = 4.8, SD = 0.6), members were able to bring their expertise to the meetings (M = 4.3, SD = 0.5), contributions were understandable and comprehensible (M = 4.6, SD = 0.5), the contributions were heard by the researchers (M = 4.6, SD = 0.7), and they added to the discussions (M = 4.1, SD = 0.3). The contributions of the different members also sometimes led to a change of one's own perspective (M = 3.7, SD = 1.0). Overall, members of the competence group assessed all quality aspects of their involvement slightly better than the advisory board members did; especially being heard by the researchers (competence group: M = 5.0, SD = 0.0, advisory group: M = 4.4, SD = 0.7).

The collaboration between the members of the advisory board and the competence group and the researchers was assessed slightly mixed (Figure 2B). Respondents were rather indifferent whether their recommendations were considered (M = 3.8, SD = 0.7) and ultimately implemented by the research groups (M = 3.6, SD = 0.5).

Overall, respondents were very satisfied with the structure of the panels (M = 4.4, SD = 0.5) as well as with the development of the research groups (M = 4.3, SD = 0.6), and strongly recommended to participate in such panels (M = 4.6, SD = 0.7).

When asked for their overall impression of their work in the panels in the open-ended question, respondents stated that they learnt a lot and enjoyed being part of the research projects.

### Interviews

To gain more in-depth insights, thoughts, and reasons about how stakeholders perceive transdisciplinary collaboration with researchers, we conducted semi-structured interviews. Five interviews were conducted: namely with two competence group members (i.e., children of parents with mental illnesses) and three advisory board members (one adult who lived with a parent with mental illness in the childhood, one expert in open innovation in science, one expert from the field of psychology).

#### The Role of the Advisory Board and the Competence Group

The various members perceived their roles very differently: For the competence group members, it was clear from the beginning what role they would play. The fact that they were asked to work as children of a mentally ill parent for the two research groups, was a sign of incredible appreciation for them: appreciation not only for a Research Topic that was so dear to their heart but also appreciation of their very personal, lived experience that could enrich research in many multi-faceted ways. The two projects were “…* for someone who is affected a sign of incredible appreciation, because you see the issue that is important to you and with which you, as an affected person identify is being taken up, is seen, money is put into it, something is set in motion*.” The competence group members aimed to shape the research as actively as possible so that on the one hand scarce resources (particularly, money and personnel) were used most effectively and on the other hand that as much research as possible could be done on an under-researched topic. The members aimed to bring as much experiential knowledge to the table as possible and wanted to enrich the project with constructive feedback that often turned out to be quite critical. Among the competence group members, the atmosphere was described as very harmonic and empathetic. They experienced an immediate bond between them right from the beginning due to their background as children of parents with mental illnesses.

For the advisory board members, in contrast, it was not that clear how exactly they could support the research groups. No one had a concrete idea of their role at the beginning. It took some time to figure out what each member could contribute to the research groups. “*You don't necessarily have an idea at the beginning. You enter a new setting, which was developed by the open innovation in science [approach] itself. And you first have to orient yourself, so to speak. You try to find out, so to speak, what my role could be. What can I contribute in relation to other participants?*” Over time, however, everyone grew into his/her role. All members aimed to support the researchers as much as possible and to give them constructive and helpful advice—without judging any of their decisions as right or wrong, or good or bad. The fact that the advisory board itself was multidisciplinary was seen as a particular enrichment: The members felt it important to share their perspectives, and to bring their experience and skillset to the table even if it was sometimes quite challenging to funnel the various input and expectations for the projects into specific recommendations. Generally, the disciplinary differences and the different expectations of the advisory board members about what research should achieve resulted in some disagreement among themselves. Nonetheless, these differences gave rise to mutual learning and richness for the whole process.

#### Collaboration With Researchers

As to the collaboration with the researchers, the competence group members perceived great insecurity on the part of the researchers on how to interact and work with them. Researchers seemed to no have an idea how the collaboration with a competence group should look like. “*Some researchers were like clumsy puppies trying to grasp us. Who are they? How do I deal with them? Also fears, fears of contact.”* Therefore, in the beginning, different forms of collaboration developed. “*Some researchers were very open and very appreciative of the competence members' experiential knowledge from the beginning; they actively asked for feedback and carefully listened to the competence members' feedback. Others were more at loss what to do with the competence group, just told the group what they planned to do without asking for feedback, and overall gave the impression that the competence group members first needed to prove themselves and their value to the project and the research*.” “*Some were able to perceive this more as constructive for themselves and as enrichment. And for others, it was the case that the perception of others, the evaluation and the defense played a greater role and that one then insisted more strongly on emphasizing the autonomy of the researcher*.” “*It really depended on the personality of the researchers to what extent they were able to accept feedback*.”

Competence group members also reported that, over time, both sides started to realize that they could learn a lot from each other due to their various backgrounds, trainings, and skillsets, and that seriously and actively engaging the competence group was an incredible benefit. Particularly for the methodological design and data analysis, the perspectives and the experiential knowledge of the competence group seemed to enrich the projects tremendously. “*We discussed the data analysis and afterwards the researcher was really happy and completely flabbergasted. She said that she had a problem with the data because the data were contradictory for her. And we were all able to say unanimously that's completely logical and gave examples and then she was like: that makes so much sense now. And I thought, yes, that's exactly why I think it's important that we are involved in the data analysis*.” However, the competence group members remained unsure until the end to what extent their feedback was indeed taken seriously and acted upon, and it was not just pure lip service from the researchers that the competence group's advice and feedback were valuable to the projects.

The advisory board members experienced the collaboration with the researchers as a balancing act. While the members always aimed to support the researchers in their plans so that they made progress and those resources were used efficiently and effectively, some researchers interpreted their advice and feedback as interference with their autonomy. It took a period of mutual learning from each other's expertise to reach a shared understanding of the conceptual frameworks and foci of the research projects. Altogether, the collaboration between the advisory board members and the researchers was considered productive and helpful. In retrospect, advisory members yet wished for more time and effort on the part of the researchers to establish and work on their relationships. “*I actually wish that we had been able to be more helpful to the project and I think that would have required more ongoing contact. It would have required the project team to have the time and effort to put into establishing and working in those relationships*.”

#### Joint Development of Approaches

Altogether, the competence group members were very open to jointly develop research approaches together with the researchers. At the same time, however, they were quite uncertain how much they were allowed to get involved into the project and particularly how much they were allowed to propose alternatives and changes to the planned research course since the basic research agenda was already defined in the Ideas Lab. How the joint development of research approaches exactly looked like, was dependent on the researcher. Competence group members reported that some researchers more strongly thought about deliverables and publications as the ultimate goals and involving the competence group was then more a box ticking exercise. Other researchers seemed to have an authentic interest in getting to know and integrating the perspectives of children of parents with mental illnesses. “*Some researchers thought only of their deliverables. Others went more into interaction and also showed: I am an interested researcher and I want to learn something from you and get something out of you. I want to experience your world*.”

The advisory board members were very open and interested in supporting and giving advice to the researchers regarding research approaches. Bringing the people together despite the internationally different time zones in which the members were located was sometimes quite challenging. In one of their meetings with the researchers, the advisory board members proactively addressed the issue of how they could be more helpful to the researchers and suggested that discussing specific questions that researchers had would be more productive than just being presented with what the researchers had been working on. While the advisory board members overall valued the whole process of being engaged and felt that their meetings were productive, they still had the impression that researchers could have reached out a little more. “*One of the things we brought up in one of our conversations was how can we be more helpful, are there specific issues that need to be addressed or specific questions that you have that we would be able to help you answer. I actually think the project team could have reached out a little more in that regard. I wish there had been more and better ways for us to be more helpful*.” Until the end, advisory board members were never quite certain whether and to which extent researchers followed up on their recommendations: “*I think we gave them good advice. It's not clear to me that they ever took our advice*.” The members reported that they never received feedback on which piece of advice worked and which piece did not work. They felt that this would have required more communication, time, and effort on the researchers' part. At the same time, however, the advisory members admitted that they never actively solicited this type of communication.

#### Integration of Different Perspectives

When it came to the integration of different perspectives, the competence group members always got the feeling that their perspectives and their experiential knowledge were heard, considered, and implemented. The members also reported that actual feedback loops were missing. The members felt that researchers did not update them in the meetings whether and to what extent their feedback was indeed implemented. However, they also admitted that they never actively asked for feedback loops. They also reported that it took them a lot of energy to make themselves heard and to convince researchers that their experiential knowledge also counts and not only the researchers' formally learned knowledge. “*It also took energy to keep pointing the finger and being critical. And I think that also has a lot to do with values. Without a title, I don't really have much of a say in this whole machinery with my expertise, which is ‘only' based on personal experience. It doesn't have the same status*.”

The advisory board members felt that openness to integrate different perspectives in their work very much varied among the researchers. Some researchers seemed to present their work as already on track, so no advice was needed from the advisory board. Other researchers seemed to perceive the advice from the advisory board as unjustified criticism of their work and interference with their work and were not willing to engage with the advisory board on a profound level. Other researchers in turn were extremely open toward the feedback of the advisory board and valued their perspective from the outside. The members reported that researchers also opened up about the various challenges (i.e., staffing, budget, administration) that they were facing on a day-to-day basis, which in turn helped the advisory board to better understand specific decisions and approaches from part of the researchers, and ultimately helped the advisory board to give advice that was more helpful. “*I feel like I had respect for and an understanding of people's different perspectives. You need to choose people with diverse expertise but who are open to different perspectives, and who are willing to brainstorm about different the application of different perspectives, and what that suggests in terms of recommendations and outcomes*.” Overall, the advisory board members felt that their success was very much dependent on how the researchers perceived the board's role and their advice and how much the researchers themselves were willing to listen and reflect.

#### Challenges for Researchers

From the perspective of the competence group, the biggest challenge the researchers were facing in their transdisciplinary work was the integration of the various perspectives and skillsets. Not only the researchers brought different trainings, perspectives, and skills to the table. Also, the members of the competence group were just not only children of parents with mental illnesses, but they also brought professional trainings and resources with them. This heterogeneity of trainings, perspectives, and skills was a huge enrichment for the research process but made everything also more complex. “*This transdisciplinarity is the work of the now and the future. These many perspectives that come in. They're a huge enrichment; they also make it more complex, of course. Because I go far beyond the level of content*.”

Advisory board members named complexity as the biggest challenge for researchers. Specifically, the biggest obstacles were the complexity to integrate the various perspectives and to agree to a research agenda that everyone could support. It was only when researchers began to communicate these difficulties honestly and openly that the advisory board members felt that they could give good advice. This kind of open and honest communication about research challenges fostered mutual learning on all sides, according to advisory board members. Advisory board members also encouraged the researchers to be courageous, to meet the challenges head on and to not try to do everything perfectly: “*You can simply be courageous. The challenges and the problems that arise, and also to enter into the debate. And not to think that I have to sweep it under the carpet, that everything has to be perfect and so on. That was always my role, to be honest and open, and then others can get on board and learn something from you*.” From the advisory board's view, the competence group presented another line of conflict. As research was done on one's own problem, debates about the adequate research process were often highly emotional.

#### Differences to Traditional Approaches

The competence group members felt that—compared to traditional research approaches—the transdisciplinary collaboration allowed a much more inclusive process. Researchers and competence group members seemed to mutually learn from each other and influence each other so that the research projects could indeed exert enduring and positive impact on the lives of children with mentally ill parents.

The advisory board observed five major differences that uniquely distinguished the transdisciplinary approach from more traditional research approaches: variety of perspectives, flexibility, community work, impact, and boldness of the funder. The members reported that firstly, the variety of perspectives arose not only from the transdisciplinary team of researchers but also the transdisciplinary nature of the competence group and the advisory board. Many different perspectives, trainings and skillsets enriched the whole research process in various ways. Secondly, the transdisciplinary approach allowed a flexibility to reflect on the whole research process and to adapt goals and methods along the way. Advisory board members were certain that such kind of flexibility would not have been possible in traditional research approaches where researchers worked through the work packages as they were described in the research proposal. Thirdly, advisory board members felt that involving children of parents with mental illnesses in the research process laid the foundation for community work. Researchers went out to the communities to involve the various stakeholders and to integrate their perspectives. Researchers themselves seemed to learn from the communities in an iterative process. “*Community work is so much harder and takes so much more time and is so much more challenging. So, the metrics that you use for evaluating success of this initiative need to reflect the fact not only that it's the open innovation business, but also that it's so community-based*.” Fourthly, advisory board members reported that the impact that a research project using a transdisciplinary approach could have, was very different from the impact that traditional research had. Not the number of published papers or the number of citations counted, but how many people had been touched by the research projects mattered: “*you may need to think carefully about things like number of people's lives you've touched, number of kids involved, number of kids who participated in making the project happen, number of families who have been touched in some way, number of other kinds of stakeholders/providers. You may want to think of your social media posts and the volume of likes or shares*.” Fifthly and lastly, the advisory board members mentioned the boldness of the funder to not only provide substantial funding for projects that have never been carried out in this way, but also to provide strong organizational support that accompanied the projects.

#### Enablers and Barriers for Successful, Transdisciplinary Collaboration

As to successful transdisciplinary collaboration, the competence group members named various enablers and barriers. Firstly, competence group members reported that transdisciplinary collaboration needed regular exchange with the whole group. Sometimes the competence group only met with some researchers but not the whole research group, which led them to focus too much on details and lose sight of the big picture. In connection to this, the competence group recommended children of parents with mental illnesses as co-researchers who were actively involved in the research process. In doing so, these experts would not be seen as some foreign parts loosely attached to the research but as a permanent and equal part of the research team itself. Thirdly, competence group members felt that transdisciplinary collaboration needed a connector—a person positioned between children of parents with mental illnesses and the researchers, who spoke both languages, knew how to mediate the different perspectives, and was convinced that transdisciplinary collaboration benefited research and society. “*For me it was a key person in the process, an excellent link between the structures. And I think the format always needs someone who carries it and who carries the format with him and says, this is so important, I live this authentically and embody this*.” The fourth enabler for successful, transdisciplinary collaboration between researchers and stakeholders that competence group members mentioned was an open mindset. All people involved along the various research stages were asked to have an open mindset. They needed to be open-minded to engage with each other, to learn from each other and to accept that sometimes research does not evolve as planned and approaches need to be adapted. Lastly, transdisciplinary collaboration needed quick wins: rapid results that were tangible for those affected so that they could see that researchers made progress, and that progress positively affected their lives.

The advisory board members also mentioned that having children of parents with mental illnesses as co-researchers in the research team would certainly promote transdisciplinary collaboration. In addition, an open, flexible, and creative mindset contributed to the success of such collaboration according to advisory board members. Everybody involved needed to be open-minded toward new perspectives and approaches, flexible to adapt the research process along the way, and creative in dealing with the different perspectives, trainings, and skills that everyone brings to the table. Additional enablers for successful, transdisciplinary collaboration from the perspective of the advisory board were early involvement, relationship management, and alternative dissemination forms. The advisory members suggested that everybody who needed to be involved in the research project should be involved as early as possible. In fact, already in the Ideas Lab those affected should be involved so that they could gain an understanding and insights into how the idea for the specific project developed.

Furthermore, relationship management was mentioned as an indispensable pillar for transdisciplinary collaboration. As the various members of the research team, the advisory boards, and the competence groups hardly knew each other at the beginning, relationships needed to be built via social events and by sharing information and communicating with each other as much as possible. “*Relationships matter and communication matters and information sharing matters. Some of the biggest challenges have been around this issue of communication and sharing information*.” Lastly, advisory board members reported that research results should not only be disseminated via the traditional ways like publications and conference presentations but also via new and innovative ways that most likely reached those concerned, for instance via community outreach events.

Members also reported that successful, transdisciplinary collaboration started with a clear commitment of the organization to support the transdisciplinary structure accompanied with the boldness to sufficiently fund such research and a dedicated person who managed knowledge and workflows between researchers and competence group and advisory board. Additionally, right from the beginning, everyone involved (i.e., researchers, members of the competence group and the advisory board) needed to be aware of what to expect from each other, and what trainings and skillsets everyone could bring to the table. Regarding the specific collaboration between researchers and competence group and advisory board members, the definition of some ground rules (like, how and when to ask for feedback) might become beneficial for productive, transdisciplinary collaboration. In this way, misunderstandings—particularly when it comes to advising researchers vs. interfering with research plans—can be eliminated right from the start.

Overall, stakeholders felt that successful, transdisciplinary collaboration between them and researchers was dependent on the researchers' attitudes. Researchers needed to be open-minded toward new perspectives and approaches, flexible to adapt the research process along the way, and creative in dealing with the different perspectives, trainings, and skills. Additionally, open, honest, and regular communication about day-to-day challenges that researchers were facing fostered mutual learnings and helped competence group and advisory board members to give advice that was more helpful. Table 1 summarizes the enablers and drivers for successful, transdisciplinary research approaches.

**Table 1:** Summary of enablers for successful, transdisciplinary collaboration.

**Domain**	**Enablers**	**Examples from interviewees**
Governance	Commitment and boldness of funders	…for someone who is affected a sign of incredible appreciation, because you see the issue that is important to you and with which you, as an affected person identify is being taken up, is seen, money is put into it, something is set in motion.
	Supporting interactions	For me it was a key person in the process, an excellent link between the structures. And I think the format always needs someone who carries it and who carries the format with him and says, this is so important, I live this authentically and embody this.
	Openness and flexibility to adaptations	You enter a new setting, which was developed by the OIS itself. And you first have to orient yourself, so to speak. You try to find out, so to speak, what my role could be. What can I contribute in relation to other participants?
Collaboration	Open-minded personality	They went more into interaction and also showed: I am an interested researcher and I want to learn something from you and get something out of you. I want to experience your world.
	Relationships and communication	Relationships matter and communication matters and information sharing matters. Some of the biggest challenges have been around this issue of communication and sharing information.
	Insecurities and tensions	Some researchers were like clumsy puppies trying to grasp us. Who are they? How do I deal with them? Also fears, fears of contact.
	Appreciation of different perspectives	I feel like I had respect for and an understanding of people's different perspectives. You need to choose people with diverse expertise but who are open to different perspectives, and who are willing to brainstorm about different the application of different perspectives, and what that suggests in terms of recommendations and outcomes.
	Feedback loops required	Constant feedback rounds were needed […] I always tried to give very hones feedback […] only positive feedback is often too little, especially in an area where so much has to happen when it comes to involving people who have experience with it.
Challenges	Heterogeneous backgrounds and skills	This interdisciplinarity is the work of the now and the future. These many perspectives that come in. They're a huge enrichment; they also make it more complex, of course. Because I go far beyond the level of content.
	Complexity	You can simply be courageous. The challenges and the problems that arise, and also to enter into the debate. And not to think that I have to sweep it under the carpet, that everything has to be perfect and so on. That was always my role, to be honest and open, and then others can get on board and learn something from you.
Impact	Community work	Community work is so much harder and takes so much more time and is so much more challenging. So, the metrics that you use for evaluating success of this initiative need to reflect the fact not only that it's the open innovation business, but also that it's so community-based. You may need to think carefully about things like number of people's lives you've touched, number of kids involved, number of kids who participated in making the project happen, number of families who have been touched in some way, number of other kinds of stakeholders/providers. You may want to think of your social media posts and the volume of likes or shares.

## Discussion

Working collaboratively and openly in a transdisciplinary research environment brings a range of challenges. In this study, we reported how stakeholders perceive transdisciplinary collaborations with researchers. Furthermore, we highlighted the enablers and barriers for such collaborations from the viewpoint of stakeholders.

### Governance Structure

Overall, the advisory board and competence group perceived the general set up, such as the duration, the frequency, preparation material and the facilitation of the panel meetings, very positively. More importantly, they reported that the structure bringing together different expertise and perspectives caused challenges and resulted in more time-consuming decisions in the panel meetings (Figures 1A,B). These aspects well-reflect the considerations of practical support as enablers of PPI (45). The competence group especially emphasized these aspects probably due their (experiential) experience and their limited knowledge of the research process. Similar patterns can also be seen in sandpit approaches, where participants described that “the social dynamics are as interesting as the science” (50). The “language of collaboration” and building trust that makes it easier to challenge different perspective needs to be established before digging into content-related discussions (51).

Competence group and advisory board members rated the quality of involvement interacting with researchers high. This is in line with reports on high levels of consensus among stakeholders regarding the added value and impact of PPI in research (34, 52). However, the collaboration between the members and the researchers and implementation of recommendations was assessed mixed (Figures 2A,B). This might be due to the barriers of PPI (45), which could either result in an tokenistic attempt if the PPI principles are not met (30, 53), or in failure to involve the public meaningfully, which may result in an unsuccessful collaboration with the public due to negative attitudes held by researchers (39).

### Enablers and Barriers on the Organizational Level

The interviews revealed several enablers for a successful, transdisciplinary research approach on two levels: the organizational (governance) and the individual level (summarized in Table 1). This ties to existing research on the principles for stakeholder engagement which can be organized in organizational factors, values and practices (54).

The advisory board emphasized the funders' commitment and boldness as an important factor to enable such a transdisciplinary approach. This is in line with other studies that mentioned financial and general support and resources, and the organizational commitment as key barriers of PPI in health research (41, 42, 45). In fact, LGB invested more than six million Euros in the entire bottom-up approach: from setting the research priority with the community to implementing the innovative research approaches for COPMI where the community defined the Research Topic and stayed an integral part in the research process along the implementation. This transdisciplinary research approach ensures that these areas can and are appropriately funded and staffed by talented individuals who want to dedicate their creative scientific talents to broader issues than their own field in the long term (55).

Furthermore, the interviewees indicated that organizational support structures, such as a person facilitating and supporting the community and stakeholder interactions, links the governance structures and acts as a key player in the process. Similarly, other studies reported the importance of support on an emotional, financial and practical level that is needed for involved people [e.g., see review (45)]: for example, support with the timing of activities, setting and constraints and commitment of public members, providing mentoring and a supportive chair to implement PPI practices. Researchers described the significant additional administrative labor and the lack of practical support for their work, as well as the time and effort diverted from these activities as barrier of PPI (19). Such a key person acts as a contact person for researchers and stakeholders and ensures that support is provided on an organizational, value-based and practice level. For example, the person fosters shared commitment to values and objectives of stakeholder engagement in the project team, recognizes potential tensions between productivity and inclusion, and considers how input from stakeholders can be collated, analyzed and used (54). In line with that, the competence and advisory group members emphasized the importance of such a key player in the process and, in fact, a relationship manager was established for the research groups Village and DOT. However, this person was placed at the LBG headquarters and not at the research groups' local site. Many difficulties arose due to this structure: for example, extensive travel time in setting up stakeholder and community relations at the beginning of the project, not being part of the research team and therefore ongoing negotiation of the roles and tasks as well as less involvement in discussions and decisions. These circumstances led to a change of the role over the years: from a relationship manager (active) to a sparring partner (passive) who discussed the progress of the research groups. One solution—as also indicated by our findings—could be to install a liaison between researchers and people with lived experience who facilitates and supports interactions between the two communities locally. In line with that, the LBG have recently begun to experiment with a new governance structure by embedding a local “stakeholder relationship manager”. This manager facilitates the interaction between stakeholder groups and researchers. Another enabler for successful, transdisciplinary collaborations is to embed people with lived experience (in our case COPMIs) as co-researchers in the research team, which has also been suggested by the advisory board and competence group members. The latter even underlined that the involvement as co-researchers would devote the necessary time, commitment, and honorarium of contributions. Further it requires an understanding of the involvement process and to create a “real” position in the research team that had been described previously (19, 30). This addition to the governance structure would involve people with lived experience early right on from the beginning and in each phase of the research process. The advisory board hereby also suggested to involve everybody who needs to be involved as early as possible, in fact, already in the Ideas Lab to gain understanding and insights. These outlined modifications in research teams might ultimately overcome frictions in relationships between researchers and stakeholders and shift power dynamics (42, 45). Working as co-researchers guarantees mutual respect and equality between researchers and the public, and might rebalance the relationship and roles. Eventually, co-researchers might foster active involvement of stakeholders in health research (39).

### Enablers and Barriers on the Individual Level

On the individual level, we also identified enablers and barriers for transdisciplinary collaboration between researchers and stakeholders. One major enabler for a successful, transdisciplinary research approach are the researchers' attitudes and values toward patient and public involvement (39, 43). Stakeholders mentioned as a crucial mindset that researchers need to bring to the table: open-mindedness, appreciation for stakeholders, eagerness to learn from other people's perspectives, interest to invest in relationships, continuous communication with stakeholders to address insecurities and tensions arising in the interaction with others, to provide feedback and actions based on the recommendations, respect for heterogeneous backgrounds and skills, and handling of complexity in an honest and open way. Previous studies explored health researchers' attitudes toward PPI and identified the transferring and sharing of power and the misconception of PPI—as participation in clinical trials and dissemination of information and knowledge—as major barriers for successful implementation (39, 40). The latter has been also reported in a recent study (56) that reflects on the limited PPI practices in Austria.

These enablers are also in line with the personal attitudes and values required for participating in the Ideas Lab (51). Based on researchers' attitudes and values captured in the application forms, only researchers describing a positive approach to team work, collaborative working and working with different disciplines and stakeholders were invited to participate in the Ideas Lab. However, these attitudes and values are often not lived and embodied in “real” collaborations with the community. Guimaraes et al. (44) explored the characteristics of inter- and transdisciplinary researchers. The authors found a mix of motivations, attitudes, skills, and behaviors, such as a humble attitude toward the immensity of knowledge, openness to different types of knowledge, tolerance to ideas opposed to one's own view, self-reflectiveness and curiosity, the ability to think in a complex and interlinked manner, and good communication and listening skills. However, these attitudes often do not link to the academic environment and its career paths, where short-term contracts and funding deadlines challenge researchers' ability to involve the public (39). Furthermore, responsibility among researchers is not distributed equally as often female researchers and early career researchers are tasked with stakeholder involvement. Ultimately, these circumstances cause tensions for those who (try to) acknowledge the value of PPI. Not surprisingly, researchers' attitudes toward PPI range from cynical to ambivalent to excited (19). Researchers further reported feelings of concerns when applying PPI practice, which may be due to a natural response to change. They also expressed concerns that PPI undermines professional skills and academic knowledge leading to a sense of de-professionalization (39). Furthermore, in this study, advisory board and competence group members reported indicated that researchers with a positive mindset and values toward PPI dealt with uncertainties and tensions better than researchers who embodied a more traditional scientific approach. To overcome this barrier, the competence group members suggested to organize social events and opportunities to meet outside the research context.

According to our results, it seems that flexibility and creativity are beneficial skills to deal with the challenges and the complexity that arise from transdisciplinary work, to change research approaches and to react to stakeholders' needs. This in turn requires to respect and appreciate heterogeneous backgrounds, different perspectives, professional trainings, and skills that all eventually enrich the discussions and collaborations (19, 44, 45). It therefore is important to carefully reflect on the who and why of involving people with lived experience so that ineffectiveness, tensions, and tokenistic involvement of stakeholders can be avoided (38).

The advisory board also emphasized different dissemination strategies to better highlight the impact that research has on the community, and alternative ways to measure scientific impact (32). Equally important is continuous communication and feedback loops about the implementation of recommendations; a crucial point that has also already been addressed in public involvement guidelines for researchers [e.g., see (57)].

Based on our results, it becomes evident that successful, transdisciplinary collaboration demands specific personality characteristics (44), organizational and financial support structures (45) and highly depends on the peoples' attitudes and values toward PPI (37, 39, 41, 43, 52). Understanding the situational context and the people and the community in which the collaboration takes place (36–38), is crucial; especially for solving complex challenges where multiple stakeholders are involved, such as designing interventions for COPMIs and their families (14–18). Our findings therefore contribute to implementation strategies, in which COPMIs have a key role in recruiting and training researchers with a positive attitude toward PPI and transdisciplinary collaboration, and in identifying tensions in the transdisciplinary collaborations.

### Strengths and Limitations of This Study

A strength of this study is that it analyzes for the first time how stakeholders perceive transdisciplinary collaboration; specifically, what enablers and drivers for such collaborations stakeholders can identify. In doing so, our study adds further evidence to previous studies that highlighted how researchers themselves can influence the success of transdisciplinary collaboration. Additionally, and also in line with previous studies, our findings underline the importance of a “neutral” contact person who facilitate the collaboration process between stakeholders and researchers, who addresses uncertainties and tensions, and who mediates among the people involved.

On a methodological level, a limitation of this study concerns the small sample size of the survey. While the competence groups and advisory boards comprised 18 people in total, 11 members responded to the survey. Therefore, we analyzed the data descriptively. To counteract any possible biases, the semi-structured interviews were conducted by a researcher working at LBG, who did not have previous contact or worked with the research groups or advisory board members before. However, it cannot be ruled out that some biased still emerged. Another limitation of this study is that we did not incorporate the researchers' perspective. After careful consideration, we decided not to invite researchers to participate in the survey and the interviews because of the upcoming evaluation of the research groups at the end of 2021 and the already existing literature on researchers' attitudes and vales toward PPI (39, 44, 45, 52). We rather wanted to focus more strongly on the stakeholders' views on transdisciplinary research collaboration.

## Conclusion

The new governance structures comprising transdisciplinary expertise and children of parents with mental illnesses was highly appreciated among the advisory board and competence group members and added value to the discussions about real life-problems and novel research approaches for COPMI. The transdisciplinary collaboration demanded a thorough understanding of people's perspectives, investment in relationships, and continuous feedback and communication with stakeholders. Furthermore, advisory board and competence group members suggested to continuously invite people with lived experience (in this case, COPMIs) as co-researchers. Open-mindedness toward different perspectives and approaches, flexibility to adapt to the research process along the way, and creativity dealing with other backgrounds and skills were identified as the most important enablers for a successful, transdisciplinary research approach. Consequently, we can conclude that peoples' attitudes and values as well as support structures are key enablers for transdisciplinary research approaches. In our experience, researchers who acknowledge the benefit of PPI practices and have already gained positive experiences working with people with lived experience (COPMI) and stakeholders are more likely to value transdisciplinary collaborations.

Future studies should aim to develop a deeper understanding of attitudes and values work as barriers for transdisciplinary collaborations between researchers and stakeholders. Specifically, future studies should focus on openness as a key enabler for transdisciplinary collaborations and might therefore answer a question that this study has unveiled. To what extent and how is it possible to create awareness and an open mindset among researchers—for instance, via capacity building and trainings—so that transdisciplinary research approaches can successfully be implemented in the future?

## Data Availability Statement

The raw data supporting the conclusions of this article will be made available by the authors, without undue reservation.

## Ethics Statement

Ethical review and approval was not required for the study on human participants in accordance with the local legislation and institutional requirements. The patients/participants provided their written informed consent to participate in this study.

## Author Contributions

RK organized the Ideas Lab in 2017 and facilitated the implementation of the LBG Research Group DOT and Village as relationship manager (2018–2021). RK and CG conceived and planned the online survey for the competence group and advisory board members and contributed to the interpretation of survey findings and interviews, conceptualization of this article, and wrote the first manuscript draft. CG designed the online survey in Unipark, which was then administered by RK. Survey results were analyzed by CG. CG also conducted the semi-structured interviews of competence group and advisory board members. All authors critically reviewed the manuscript for intellectual content, approved the final version, and agreed to be accountable for all aspects of the work in ensuring that questions related to the accuracy or integrity of any part of the work are appropriately investigated and resolved.

## Funding

The open innovation initiative and following projects are funded by the LBG Open Innovation in Science Center at the Ludwig Boltzmann Gesellschaft, Austria.

## Conflict of Interest

The authors declare that the research was conducted in the absence of any commercial or financial relationships that could be construed as a potential conflict of interest.

## Publisher's Note

All claims expressed in this article are solely those of the authors and do not necessarily represent those of their affiliated organizations, or those of the publisher, the editors and the reviewers. Any product that may be evaluated in this article, or claim that may be made by its manufacturer, is not guaranteed or endorsed by the publisher.

## References

[1] Maybery D Reupert A Goodyear M Ritchie R Brann P. Investigating the strengths and difficulties of children from families with a parental mental illness. Austral E J Adv Mental Health. (2009) 8:165–74. 10.5172/jamh.8.2.165

[2] Maybery D Reupert AE. The number of parents who are patients attending adult psychiatric services. Curr Opin Psychiatry. (2018) 31:358–62. 10.1097/YCO.0000000000000427 29847344

[3] Ostman M Hansson L. Children in families with a severely mentally ill member. Prevalence and needs for support. Soc Psychiatry Psychiatr Epidemiol. (2002) 37:243–8. 10.1007/s00127-002-0540-0 12107717

[4] Pretis M Dimova A. Vulnerable children of mentally ill parents: towards evidence-based support for improving resilience. Support Learn. (2008) 23:152–9. 10.1111/j.1467-9604.2008.00386.x

[5] Hosman CMH van Doesum KTM van Santvoort F. Prevention of emotional problems and psychiatric risks in children of parents with a mental illness in the Netherlands: I. The scientific basis to a comprehensive approach. Austral E J Adv Mental Health. (2009) 8:250–63. 10.5172/jamh.8.3.250

[6] Power J Goodyear M Maybery D Reupert A O'Hanlon B Cuff R. Family resilience in families where a parent has a mental illness. J Soc Work. (2016) 16:66–82. 10.1177/1468017314568081

[7] Reupert A Maybery D. Families affected by parental mental illness: a multiperspective account of issues and interventions. Am J Orthopsychiatry. (2007) 77:362–9. 10.1037/0002-9432.77.3.362 17696664

[8] Reupert AE Cuff R Drost L Foster K van Doesum KT van Santvoort F. Intervention programs for children whose parents have a mental illness: a review. Med J Aust. (2013) 199(3 Suppl.):S18–22. 10.5694/mja11.11145 25369843

[9] Goodyear M Hill TL Allchin B McCormick F Hine R Cuff R. Standards of practice for the adult mental health workforce: meeting the needs of families where a parent has a mental illness. Int J Ment Health Nurs. (2015) 24:169–80. 10.1111/inm.12120 25619407

[10] Lauritzen C Reedtz C Van Doesum KT Martinussen M. Implementing new routines in adult mental health care to identify and support children of mentally ill parents. BMC Health Serv Res. (2014) 14:58. 10.1186/1472-6963-14-58 24507566 PMC4015279

[11] Maybery D Jones R Dipnall JF Berger E Campbell T McFarlane A. A mixed-methods study of psychological distress following an environmental catastrophe: the case of the Hazelwood open-cut coalmine fire in Australia. Anxiety Stress Coping. (2020) 33:216–30. 10.1080/10615806.2019.1695523 31752536

[12] Nicholson J Albert K Gershenson B Williams V Biebel K. Family options for parents with mental illnesses: a developmental, mixed methods pilot study. Psychiatr Rehabil J. (2009) 33:106–14. 10.2975/33.2.2009.106.114 19808206

[13] Solantaus T Toikka S. The effective family programme: preventative services for the children of mentally ill parents in Finland. Int J Mental Health Promot. (2006) 8:37–44. 10.1080/14623730.2006.9721744

[14] Chambers DA Glasgow RE Stange KC. The dynamic sustainability framework: addressing the paradox of sustainment amid ongoing change. Implement Sci. (2013) 8:117. 10.1186/1748-5908-8-117 24088228 PMC3852739

[15] Greenhalgh T Wherton J Papoutsi C Lynch J Hughes G A'Court C. Beyond adoption: a new framework for theorizing and evaluating nonadoption, abandonment, and challenges to the scale-up, spread, and sustainability of health and care technologies. J Med Internet Res. (2017) 19:e367. 10.2196/jmir.8775 29092808 PMC5688245

[16] Watson DP Adams EL Shue S Coates H McGuire A Chesher J. Defining the external implementation context: an integrative systematic literature review. BMC Health Serv Res. (2018) 18:209. 10.1186/s12913-018-3046-5 29580251 PMC5870506

[17] Goodyear M Obradovic A Allchin B Cuff R McCormick F Cosgriff C. Building capacity for cross-sectorial approaches to the care of families where a parent has a mental illness. Adv Mental Health. (2015) 13:153–64. 10.1080/18387357.2015.1063972

[18] Zechmeister-Koss I Goodyear M Tuchler H Paul JL. Supporting children who have a parent with a mental illness in Tyrol: a situational analysis for informing co-development and implementation of practice changes. BMC Health Serv Res. (2020) 20:326. 10.1186/s12913-020-05184-8 32306960 PMC7168853

[19] Boylan AM Locock L Thomson R Staniszewska S. “About sixty per cent I want to do it”: health researchers' attitudes to, and experiences of, patient and public involvement (PPI)-A qualitative interview study. Health Expect. (2019) 22:721–30. 10.1111/hex.12883 30927334 PMC6737750

[20] Klein JT. Evaluation of interdisciplinary and transdisciplinary research: a literature review. Am J Prev Med. (2008) 35(Suppl. 2):S116–23. 10.1016/j.amepre.2008.05.010 18619391

[21] United Nations. Transforming Our World: The 2030 Agenda for Sustainable Development (2015). Available online at: https://sustainabledevelopment.un.org/content/documents/21252030%20Agenda%20for%20Sustainable%20Development%20web.pdf (accessed July 26, 2021)

[22] National Institute of Health Research (NIHR). Involve patients. Available online at: https://www.nihr.ac.uk/health-and-care-professionals/engagement-and-participation-in-research/involve-patients.htm (accessed July 26, 2021).

[23] Carman KL Dardess P Maurer M Sofaer S Adams K Bechtel C. Patient and family engagement: a framework for understanding the elements and developing interventions and policies. Health Aff. (2013) 32:223–31. 10.1377/hlthaff.2012.1133 23381514

[24] Church J Saunders D Wanke M Pong R Spooner C Dorgan M. Citizen participation in health decision-making: past experience and future prospects. J Public Health Policy. (2002) 23:12–32. 10.2307/3343116 12013713

[25] Coultier A. Engaging Patients in Healthcare. New York, NY: McGraw Hill Professional (2013).

[26] Hayes H Buckland S Tarpey M. Briefing Notes for Researchers: Public Involvement in NHS, Public Health and Social Care Research. Eastleigh: INVOLVE (2012).

[27] Staley K. The BMJ [Internet]. BMJ (2018). Available online at: https://blogs.bmj.com/bmj/2018/11/28/researchers-dont-know-what-theyre-missing-the-impact-of-patient-involvement-in-research/ (accessed July 26, 2021).

[28] Piil K Jarden M. Patient involvement in research priorities (PIRE): a study protocol. BMJ Open. (2016) 6:e010615. 10.1136/bmjopen-2015-010615 27221126 PMC4885285

[29] Knowles SE Allen D Donnelly A Flynn J Gallacher K Lewis A. More than a method: trusting relationships, productive tensions, and two-way learning as mechanisms of authentic co-production. Res Involv Engagem. (2021) 7:34. 10.1186/s40900-021-00262-5 34059159 PMC8165763

[30] Kaisler RE Missbach B. Co-creating a patient and public involvement and engagement 'how to' guide for researchers. Res Involv Engagem. (2020) 6:32. 10.1186/s40900-020-00208-3 32566249 PMC7301967

[31] Chudyk AM Waldman C Horrill T Demczuk L Shimmin C Stoddard R. Models and frameworks of patient engagement in health services research: a scoping review protocol. Res Involv Engagem. (2018) 4:28. 10.1186/s40900-018-0111-5 30214822 PMC6130061

[32] Staley K. Exploring Impact: Public Involvement in NHS, Public Health and Social Care Research. Eastleigh: INVOLVE (2009).

[33] Shippee ND Domecq Garces JP Prutsky Lopez GJ Wang Z Elraiyah TA Nabhan M. Patient and service user engagement in research: a systematic review and synthesized framework. Health Expect. (2015) 18:1151–66. 10.1111/hex.12090 23731468 PMC5060820

[34] Brett J Staniszewska S Mockford C Herron-Marx S Hughes J Tysall C. Mapping the impact of patient and public involvement on health and social care research: a systematic review. Health Expect. (2014) 17:637–50. 10.1111/j.1369-7625.2012.00795.x 22809132 PMC5060910

[35] Domecq JP Prutsky G Elraiyah T Wang Z Nabhan M Shippee N. Patient engagement in research: a systematic review. BMC Health Serv Res. (2014) 14:89. 10.1186/1472-6963-14-89 24568690 PMC3938901

[36] Brett J Staniszewska S Mockford C Herron-Marx S Hughes J Tysall C. A systematic review of the impact of patient and public involvement on service users, researchers and communities. Patient. (2014) 7:387–95. 10.1007/s40271-014-0065-0 25034612

[37] Staley K. “Is it worth doing?” Measuring the impact of patient and public involvement in research. Res Involv Engagem. (2015) 1:6. 10.1186/s40900-015-0008-5 29062495 PMC5598089

[38] Staley K Elliott J Stewart D Wilson R. Who should I involve in my research and why? Patients, carers or the public? Res Involv Engag. (2021) 7:41. 10.1186/s40900-021-00282-1 34127074 PMC8202960

[39] Thompson J Barber R Ward PR Boote JD Cooper CL Armitage CJ. Health researchers' attitudes towards public involvement in health research. Health Expect. (2009) 12:209–20. 10.1111/j.1369-7625.2009.00532.x 19392833 PMC5060486

[40] Vale CL Thompson LC Murphy C Forcat S Hanley B. Involvement of consumers in studies run by the Medical Research Council Clinical Trials Unit: results of a survey. Trials. (2012) 13:9. 10.1186/1745-6215-13-9 22243649 PMC3398265

[41] McKenzie A Bulsara C Haines H Hanley B Alpers K. Barriers to Community Involvement in Health and Medical Research - Researchers Perspectives on Consumer and Community Involvement in Research: A Qualitative Study. Nedlands, WA: The University of Western Australia School of Population Health; Telethon Kids; Institute and The University of Notre Dame (2016).

[42] Wilson P Mathie E Keenan J McNeilly E Goodman C Howe A. Health Services and Delivery Research. In: Research With Patient and Public Involvement: a RealisT Evaluation – The RAPPORT Study. Southampton: NIHR Journals Library (2015). 26378332

[43] Nathan S Harris E Kemp L Harris-Roxas B. Health service staff attitudes to community representatives on committees. J Health Organ Manag. (2006) 20:551–9. 10.1108/14777260610702299 17168106

[44] Guimarães MH Pohl C Bina O Varanda M. Who is doing inter- and transdisciplinary research, and why? An empirical study of motivations, attitudes, skills, and behaviours. Futures. (2019) 112:102441. 10.1016/j.futures.2019.102441

[45] Ocloo J Garfield S Franklin BD Dawson S. Exploring the theory, barriers and enablers for patient and public involvement across health, social care and patient safety: a systematic review of reviews. Health Res Policy Syst. (2021) 19:8. 10.1186/s12961-020-00644-3 33472647 PMC7816359

[46] Ludwig Boltzmann Gesellschaft. Tell Us! About Mental Health: Ludwig Boltzmann Gesellschaft. Available online at: https://www.redensiemit.org (accessed July 26, 2021).

[47] Ludwig Boltzmann Gesellschaft. Ideas Lab on, Children of Mentally Ill Parents: Ludwig Boltzmann Gesellschaft. Available online at: http://www.ideaslab.lbg.ac.at (accessed July 26, 2021).

[48] Ludwig Boltzmann Gesellschaft. DOT - The Open Door: Ludwig Boltzmann Gesellschaft. Available online at: https://dot.lbg.ac.at/ (accessed July 26, 2021).

[49] Ludwig Boltzmann Gesellschaft. Village - How to Raise a Village to Raise a Child: Ludwig Boltzmann Gesellschaft. Available online at: http://village.lbg.ac.at/ (accessed July 26, 2021).

[50] Giles J. Sandpit initiative digs deep to bring disciplines together. Nature. (2004) 427:187. 10.1038/427187a 14724604

[51] Maxwell K Benneworth P. The construction of new scientific norms for solving Grand Challenges. Palgrave Commun. (2018) 4:52. 10.1057/s41599-018-0105-9

[52] Snape D Kirkham J Britten N Froggatt K Gradinger F Lobban F. Exploring perceived barriers, drivers, impacts and the need for evaluation of public involvement in health and social care research: a modified Delphi study. BMJ Open. (2014) 4:e004943. 10.1136/bmjopen-2014-004943 24939808 PMC4067891

[53] INVOLVE. Involving Children and Young People in Research: Top Tips and Essential Key Issues for Researchers. Eastleigh: INVOLVE (2016).

[54] Boaz A Hanney S Borst R O'Shea A Kok M. How to engage stakeholders in research: design principles to support improvement. Health Res Policy Syst. (2018) 16:60. 10.1186/s12961-018-0337-6 29996848 PMC6042393

[55] Holm P Goodsite ME Cloetingh S Agnoletti M Moldan B Lang DJ. Collaboration between the natural, social and human sciences in Global Change Research. Environ Sci Policy. (2013) 28:25–35. 10.1016/j.envsci.2012.11.010

[56] Kaisler RE Kulnik ST Klager E Kletecka-Pulker M Schaden E Stainer-Hochgatterer A. Introducing patient and public involvement practices to healthcare research in Austria: strategies to promote change at multiple levels. BMJ Open. (2021) 11:e045618. 10.1136/bmjopen-2020-045618 34373295 PMC8354292

[57] Kaisler RE Missbach B. Patient and Public Involvement and Engagement in Research - A “how to” Guide for Researchers. Zenodo (2019). 10.5281/zenodo.3578321 32566249 PMC7301967

