# Supplementary material for: Enabling Transdisciplinary Collaboration: Stakeholder Views on Working With “Children With Mentally Ill Parents” Research Groups
Source: Front Psychiatry. 2021 Nov 23;12:760716. doi: 10.3389/fpsyt.2021.760716 (PMC8649715; doi:10.3389/fpsyt.2021.760716)
Supplement: Supplementary file 1 [file Table_1.DOCX]

**Supplement Material**

**Table 1. Mean ratings of Advisory Board and Competence Group members in the online survey.**

| **Questionnaire** | **Overall**  **(n=12)** | |  | **Advisory Board**  **(n=9)** | |  | **Competence Group (n=3)** | | |
| --- | --- | --- | --- | --- | --- | --- | --- | --- | --- |
|  | M | SD |  | M | SD |  | M | | SD |
| The frequency of the meetings was adequate. | 4.3 | 0.9 |  | 4.3 | 1.0 |  | 4.3 | 0.6 | |
| The duration of the meetings was adequate. | 3.9 | 0.8 |  | 3.9 | 0.8 |  | 4.3 | 0.6 | |
| The format of the meetings was adequate. | 4.1 | 1.0 |  | 3.9 | 1.1 |  | 4.7 | 0.6 | |
| The meeting preparation materials were adequate. | 4.0 | 0.6 |  | 4.1 | 0.6 |  | 3.7 | 0.6 | |
| The facilitation of the meetings was adequate. | 4.6 | 0.5 |  | 4.4 | 0.5 |  | 5.0 | 0.0 | |
| The composition of the panel was adequate for the research group. | 4.3 | 0.8 |  | 4.2 | 0.8 |  | 4.7 | 0.6 | |
| The different expertise on the panel complemented each other. | 4.5 | 0.5 |  | 4.4 | 0.5 |  | 4.7 | 0.6 | |
| The different expertise on the panel have brought challenges. | 2.8 | 1.0 |  | 3.0 | 1.1 |  | 2.3 | 0.6 | |
| The different expertise on the panel took more time to make decisions. | 3.3 | 1.0 |  | 3.6 | 0.9 |  | 2.3 | 0.6 | |
| The atmosphere in the panel was appreciative. | 4.8 | 0.6 |  | 4.7 | 0.7 |  | 5.0 | 0.0 | |
| I was able to contribute my expertise to the meetings. | 4.3 | 0.5 |  | 4.3 | 0.5 |  | 4.3 | 0.6 | |
| My contributions were heard by the other experts. | 4.6 | 0.7 |  | 4.4 | 0.7 |  | 5.0 | 0.0 | |
| My contributions contributed to the discussion. | 4.1 | 0.3 |  | 4.0 | 0.0 |  | 4.3 | 0.6 | |
| The contributions of other experts were understandable and comprehensible for me. | 4.6 | 0.5 |  | 4.6 | 0.5 |  | 4.7 | 0.6 | |
| The contributions of other experts changed my point of view. | 3.7 | 1.0 |  | 3.6 | 1.0 |  | 4.0 | 1.0 | |
| The recommendations of the panel were seen as helpful and considered by the research group. | 3.8 | 0.7 |  | 3.8 | 0.7 |  | 4.0 | 1.0 | |
| The recommendations of the panel were implemented by the research group. | 3.6 | 0.5 |  | 3.6 | 0.5 |  | 3.7 | 0.6 | |
| How satisfied are you with the structure of the panel (advisory board / competence group)? | 4.4 | 0.5 |  | 4.6 | 0.5 |  | 4.0 | 0.0 | |
| How satisfied are you overall with the development of the research groups? | 4.3 | 0.6 |  | 4.2 | 0.7 |  | 4.3 | 0.6 | |
| To what extent would you recommend others to participate in these panels (advisory board / competence group)? | 4.6 | 0.7 |  | 4.6 | 0.7 |  | 4.7 | 0.6 | |

*Note.* 5-point Likert scale (1=do not agree at all – 5=fully agree; 1=not at all satisfied – 5=fully satisfied; 1=not at all recommended – 5 = very much recommended). M = mean values, SD = standard deviation.
